# Oesophageal perforation following transoesophageal echocardiography: A case report on successful conservative management

**DOI:** 10.1016/j.ijscr.2019.07.039

**Published:** 2019-07-19

**Authors:** Nadiah Binti Rosly, Guo Hou Loo, Mohamad Aznan Bin Shuhaili, Reynu Rajan, Nik Ritza Kosai

**Affiliations:** Department of Surgery, Faculty of Medicine, The National University of Malaysia, Jalan Yaacob Latiff, Bandar Tun Razak, 56000, Selangor, Malaysia

**Keywords:** Oesophageal injury, TEE, Cervical oesophagus, Cardiopulmonary bypass

## Abstract

**Introduction:**

Transoesophageal echocardiography (TOE) is a widely used intraoperative diagnostic tool in cardiac patients, and it is considered as a safe and non-invasive procedure. However, it has its known complications, which is estimated to be 0.18% with mortality reported as 0.0098%.

Complications of TOE include odynophagia, upper gastrointestinal haemorrhage, endotracheal tube malpositioning and dental injury. One of the rarer complications includes oesophageal perforation, whose incidence is reported to be 0.01%.

**Case Presentation:**

We present a case of a 61-year-old lady with mitral valve prolapse (MVP) who underwent TOE with subsequent presentation of odynophagia with left neck swelling. An upper endoscopy examination was inconclusive; however, a contrasted computed tomography of the neck showed evidence of cervical oesophageal perforation. She was managed conservatively and discharged well.

**Discussion:**

The trauma caused by TOE probe insertion and manipulation accounts for most of the upper gastrointestinal complications. Mortality of patients associated with oesophageal perforation can be up to 20% and doubled if the treatment is delayed for more than 24 h. Mechanism of injury from TOE probe is likely multifactorial. Predisposing factors that increase the risk of tissue disruption include the presence of unknown structural pathology. Imaging studies and an upper endoscopy examination may aid in the diagnosis of oesophageal perforation.

**Conclusion:**

A high index of suspicion, coupled with a tailored, multidisciplinary approach, is essential to achieve the best possible outcome. Conservative management may be worthwhile in a stable patient despite delayed presentation. Although TOE is considered a safe procedure, physicians should be made aware of such a dreaded complication.

## Highlights

• Transoesophageal echocardiography (TOE) is considered as a safe and non-invasive procedure.
• Complications include odynophagia, upper gastrointestinal haemorrhage, endotracheal tube malpositioning and dental injury.
• One of the rarer complications includes oesophageal perforation.
• A high index of suspicion is crucial, particularly if there was a struggle in introducing the TOE probe.
• Prompt diagnosis and vital investigations are crucial in order to advocate the early management of patients.
• Conservative management may be worthwhile in a stable patient despite delayed presentation.

## 1 Introduction

Transoesophageal Echocardiography (TOE) is a crucial investigative procedure for perioperative assessment of cardiac patients. It is a relatively safe procedure but carries up to 3% risk of complications [1]. Complications of TOE include odynophagia, upper gastrointestinal haemorrhage, endotracheal tube malpositioning and dental injury [2]. One of the rarer complications includes oesophageal perforation, whose incidence is reported to be 0.01–0.09%, and is higher when TOE examination occurred during intraoperative setting [3,4] Mortality of patients associated with oesophageal perforation can be up to 20% and doubled if the treatment is delayed for more than 24 h [1]. Mechanism of injury with the TOE probe is likely multifactorial [2]. Here, we report on the successful conservative management of a 61-year-old lady with iatrogenic perforated cervical oesophagus following TOE. This case has been reported in line with the SCARE criteria [5].

## 2 Case report

A 61-year-old lady presented to us with a four days history of left-sided neck swelling associated with odynophagia. She has pre-existing atrial fibrillation since ten years back and she is on non-Vitamin K antagonist oral anticoagulant (NOAC). Before this presentation, she had undergone unsuccessful transoesophageal echocardiography (TOE) at a heart centre to investigate her mitral valve prolapse. The TOE was abandoned as they had difficulty inserting the TOE probe. At the heart centre, she was monitored for a day post procedure and was discharged well.

Upon further history, she denies having upper gastrointestinal symptoms prior to this. On clinical examination, she appears well and afebrile. There was a left anterior neck swelling measuring 4 cm x 4 cm which was tender on palpation. There were no skin changes and no cervical lymph nodes palpable. All other systemic examinations were unremarkable. Initial blood investigation showed mild leukocytosis (11.4 × 109/L), but the rest of her blood investigations were normal. We suspected a delayed iatrogenic upper oesophageal perforation secondary to her recent TOE.

**Fig. 1:**
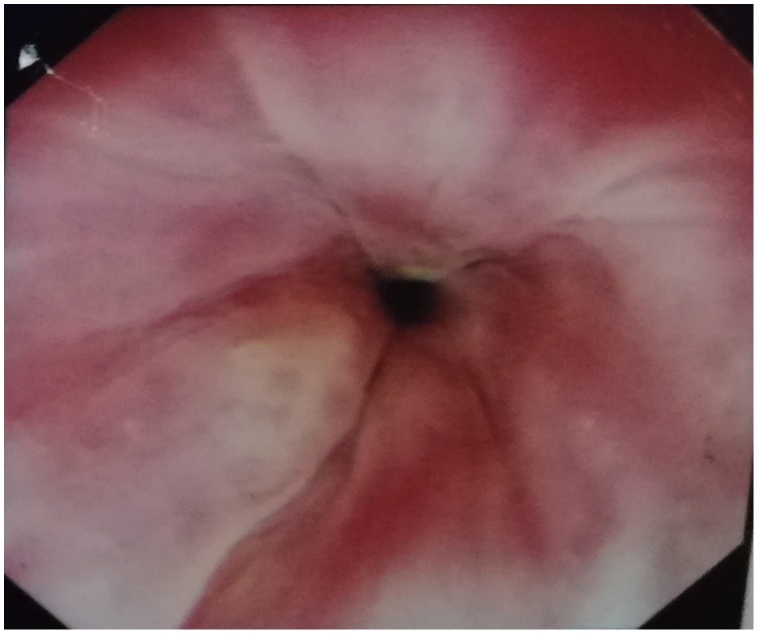
Upper endoscopy image showing normal oesophageal mucosa.

**Fig. 2:**
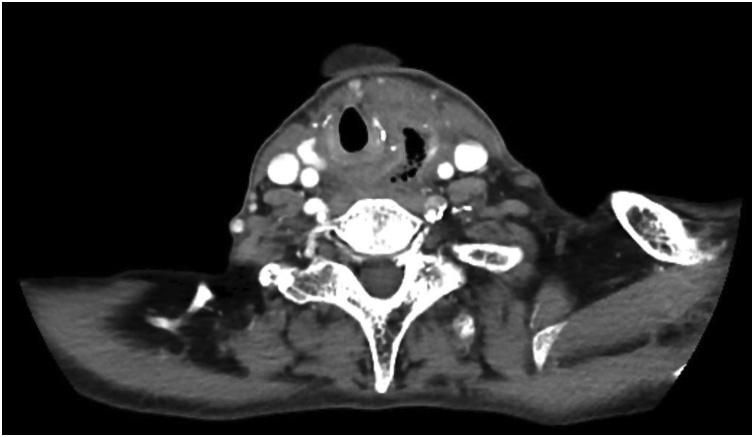
Axial view of CECT of the neck showing a prevertebral soft tissue swelling with a maximal thickness measuring 2.2 cm and the presence of air pockets at the level of C7.

**Figs. 3 and 4:**
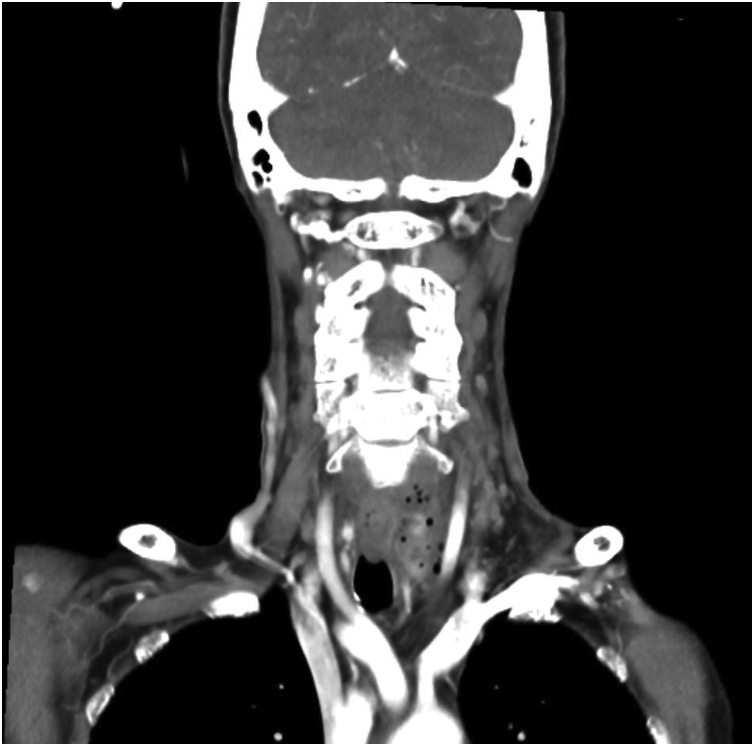
Coronal and sagittal view of CECT of the neck showing a semilunar hypodense collection with rim-enhancing wall from the left submandibular space superiorly to the suprasternal region inferiorly.

**Fig. 5:**
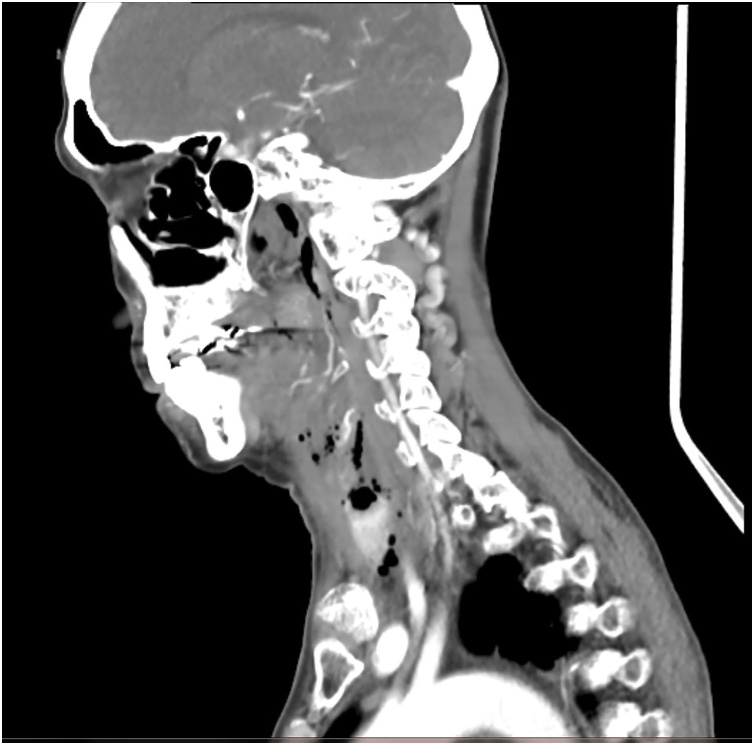
Axial view of plain CT of the neck (post gastrograffin) showing contrast leakage within the previously hypodense collection at the left paravertebral region.

We proceeded with an upper endoscopic examination, but unfortunately, we were unable to visualize any mucosal abnormalities over the oesophagus (Fig. 1). She did, however, have an associated sliding hiatus hernia. A contrasted computed tomography (CECT) of the neck was performed. It showed a prevertebral soft tissue swelling with a maximal thickness measuring 2.2 cm and the presence of air pockets at the level of C7 (Fig. 2). A semilunar hypodense collection with rim-esnhancing wall is seen from the left submandibular space superiorly to the suprasternal region inferiorly (Figs. 3 and 4 ). Subsequently, we performed a targeted upper gastrointestinal study with gastrograffin with a complementary plain CT of the neck. Contrast leakage was seen within the previously hypodense collection at the left paravertebral region, and the contrast media is seen tracking into the left submandibular space superiorly and inferiorly, until the level of T1 (Fig. 5 ). A diagnosis of oesophageal perforation is thus confirmed, although the defect is likely small, as no contrast leakage was seen during the dynamic study.

As the patient was clinically well, and the upper endoscopy examination did not show any apparent mucosal defect, we elect to manage her conservatively. She was kept nil-by-mouth for a week, and total parenteral nutrition was initiated. She was also empirically started on broad-spectrum intravenous antibiotics. Her clinical condition improved with resolution of the neck swelling as well as the odynophagia. She was started on oral feeding after a week and subsequently discharged well. She was seen in our clinic four weeks after discharge, and she remains well. A repeated upper gastrointestinal study performed six months later showed smooth contrast flow though the oesophagus with no contrast extravasation.

## 3 Discussion

Transoesophageal Echocardiography (TOE) is a crucial investigative procedure for perioperative assessment of cardiac patients. It is a relatively safe procedure but carries up to 3% risk of complications [1]. Complications of TOE include odynophagia, upper gastrointestinal haemorrhage, endotracheal tube malpositioning and dental injury [2]. One of the rarer complications includes oesophageal perforation, whose incidence is reported to be 0.01–0.09% [3,4]. Other reported serious complications include vocal cord paralysis, hypotension, dysrhythmias, seizures and cardiac arrest [1].

The trauma caused by TOE probe insertion and manipulation accounts for most of the upper gastrointestinal complications. These are usually confined to the oropharyngeal, oesophageal and the stomach [3]. Major gastrointestinal complications include oesophageal or gastric perforation, and upper gastrointestinal bleeding requiring blood transfusion, endoscopic or surgical intervention [6]. The incidence of such complications is reported to be 1.2% [5]. Late presentation (>24 h) is more common than early presentation [6]. The incidence of oesophageal perforation itself, however, is reported to be 0.03-0.09% [4] Mortality of patients associated with oesophageal perforation can be up to 20% and doubled if the treatment is delayed for more than 24 h [1].

Mechanism of injury from TOE probe is likely multifactorial [2,6] Direct trauma from the TOE probe during blind insertion and manipulation is a likely factor, especially in anaesthetized patients [2,6] Often, upper oesophageal injury or perforation occurred when the TOE operator encountered excessive resistance or difficulty inserting the TOE probe [2]. Other factors include local tissue thermal injury, pressure effects, vascular insufficiency, and local mucosal ischemia during a cardiopulmonary bypass [2,6]. Predisposing factors that increase the risk of tissue disruption include the presence of an unknown oesophageal or gastric structural pathology. These include Mallory-Weiss tear, oesophageal stenosis, Barrett’s oesophagus, Zenker’s diverticulum, tumour or strictures. Medications which may affect the integrity of oesophageal mucosae such as corticosteroids and bisphosphonates also increases the risk of oesophageal injury [2,7,8]. It is essential to note, however, that most cases of oesophageal perforation occur in patients with perceived low risk and hence, screening for predisposing risk factors may not eliminate the risk of perforation [9].

The most common site of perforation is the cervical oesophagus. It is postulated that the crossing of muscle fibres between the cricopharyngeus and the constrictor muscle of the pharynx makes this segment more susceptible to injury [8]. Similarly, in our case, the perforation occurred at the cervical oesophagus, which likely happened during probe insertion attempt. Due to the continuous movement of the cervical oesophagus from swallowing and respiration, injury or perforation to this part may give rise to significant complications. However, compared to the other segments of the oesophagus, perforation at the cervical oesophagus carries a better prognosis [10]. Cervical oesophageal perforations also tend to be detected earlier, as it usually occurs in the non-operative group of patients [9].

Symptoms of oesophageal perforation include odynophagia, painful cervical contracture, retrosternal pain, dysphagia, foul-smelling expectoration, dysphonia and dyspnea [2,8,10]. The most common clinical sign seen in cervical oesophagus perforation is the presence of subcutaneous emphysema with crepitation on palpation of the neck [10]. In the more advanced stage, cellulitic changes over the cervical region, fever and lung changes might manifest [10]. Delayed detection of an oesophageal perforation may progress to mediastinitis, multiorgan failure due to sepsis and death [4].

Prompt diagnosis and careful investigations are needed in order to advocate the early management of patients. A high index of suspicion is crucial, particularly in this patient where there was a struggle in introducing the scope. In patients who are anaesthetized after cardiac surgery, it can be very challenging to identify an oesophageal injury or perforation [4].

Imaging studies and an upper endoscopy examination may aid in the diagnosis of oesophageal perforation. Plain radiographs of the neck and chest are vital initial investigations. These will allow for the assessment of any radiopaque foreign bodies, pneumomediastinum, subcutaneous emphysema and any associated lung pathologies such as pneumothorax, effusions or consolidation [8]. However, as in our patient, chest radiographs are usually normal in patients with an oesophageal injury [4].

Upper endoscopy is a safe investigative tool in cases of oesophageal perforation [11]. The use of carbon dioxide insufflation will reduce the risk of pneumothorax or subcutaneous emphysema [11]. Contrast study of the upper gastrointestinal tract using gastrograffin or barium is also useful. Water-soluble compound is preferred as these produce minimal tissue reaction when compared to barium compound. However, false negative rates in detecting oesophageal perforation using water-soluble compounds can be as high as 50% [11]. Computed tomography (CT) of the neck when added in combination with other investigative tools, aids in localization of deep collections. It also helps in deciding whether a more aggressive or conservative approach is used, as well as for the assessment of response to treatment [11,12].

Recent evidence has shifted towards more conservative management of oesophageal perforations [13]. It compromises of keeping the patient nil-by-mouth, nasogastric suction, broad-spectrum antibiotics and parenteral nutrition. The placement of oesophageal endoscopic stents in oesophageal perforation appears to be a promising non-operative approach [11,13]. Oesophageal injury from the TOE probe is ideal cases for endoscopic stent placement as there is minimal bacterial contamination [11]. However, there are still limited data available regarding endoscopic stent placement as compared to the standard surgical approach in oesophageal perforation.

## 4 Conclusion

This case highlights the importance of early recognition and management of a rare complication of TOE. A high index of suspicion, coupled with a tailored, multidisciplinary approach, is essential to achieve the best possible outcome. This case also highlights that conservative management may be worthwhile in a stable patient despite delayed presentation. Although TOE is considered a safe procedure, physicians should be made aware of such a dreaded complication.

## Source of funding

No source of funding.

## Ethical approval

The National University of Malaysia’s Ethics Committee has exempted the need for an ethical approval for any case report being written/ published.

## Consent

Written informed consent was obtained from the patient for publication of this case report and accompanying images. A copy of the written consent is available for review by the Editor-in-Chief of this journal on request.

## Author’s contribution

Study concepts: Nik Ritza Kosai, Mohamad Aznan Bin Shuhaili, Reynu Rajan.

Study design: Nik Ritza Kosai, Mohamad Aznan Bin Shuhaili, Reynu Rajan.

Data acquisition: Nadiah Binti Rosly, Loo Guo Hou.

Quality control of data and algorithms: Nik Ritza Kosai, Mohamad Aznan Bin Shuhaili, Reynu Rajan, Data analysis and interpretation: Nadiah Binti Rosly, Loo Guo Hou.

Statistical analysis: -Not applicable.

Manuscript preparation: Loo Guo Hou, Nadiah Binti Rosly.

Manuscript editing: Loo Guo Hou.

Manuscript review: Nik Ritza Kosai, Mohamad Aznan Bin Shuhaili.

## Registration of research studies

Not applicable.

## Guarantor

Professor Dr Nik Ritza Kosai, Loo Guo Hou.

## Provenance and peer review

Not commissioned.

## Declaration of Competing Interest

No conflict of interests.
